# Effects of *Nigella sativa* oil extract on inflammatory cytokine response and oxidative stress status in patients with rheumatoid arthritis: a randomized, double-blind, placebo-controlled clinical trial

**Published:** 2016

**Authors:** Vahid Hadi, Sorayya Kheirouri, Mohammad Alizadeh, Alireza Khabbazi, Hossein Hosseini

**Affiliations:** 1 *Department of Nutrition, Tabriz University of Medical Sciences, Tabriz, Iran*; 2 *Department of Internal Medicine, Tabriz University of Medical Sciences, Tabriz, Iran*; 3 *Department of Agriculture, Barij Essence Pharmaceutical Company, Kashan, Iran*

**Keywords:** *Nigella sativa*, *Rheumatoid arthritis*, *Oxidative stress*, *IL-10*, *TNF-α*

## Abstract

**Objective:**

*Nigella sativa* is a medicinal plant that has long been used in traditional medicine for treating various conditions. Numerous animal studies provided evidences that the seed may elicit a broad anti-inflammatory/anti-oxidant activity. The aim of the present clinical trial was to evaluate anti-inflammatory and antioxidant properties of *Nigella sativa* oil in patients with rheumatoid arthritis (RA).

**Materials and Methods:**

Forty-two patients with RA were assigned into two groups in this randomized, double blind, placebo-controlled clinical trial. Subjects in intervention group received two capsules, 500 mg each, of *Nigella sativa* oil, each day for 8 weeks. The other group consumed two capsules as placebo per day for the same period of time. Serum TNF-α, IL-10, and whole blood levels of oxidative stress parameters were measured at baseline and end of the trial.

**Results:**

The serum level of IL-10 was increased in the *Nigella sativa* group (p<0.01). Moreover, treatment with *Nigella sativa* led to significant reduction of serum MDA and NO compared with baseline (p<0.05). There were no significant differences in the TNF-α, SOD, catalase, and TAS values between or within the groups, before and after the intervention (p>0.05).

**Conclusion:**

This study indicates that *Nigella sativa* could improve inflammation and reduce oxidative stress in patients with RA. It is suggested that *Nigella sativa* may be a beneficial adjunct therapy in this population of patients.

## Introduction

Rheumatoid arthritis (RA) is a disabling autoimmune disease that is characterized By significant pain, progressive joint disorder, and functional disability. Rheumatoid arthritis is a disease with unknown cause and has a prevalence of 0.5-1% among adults worldwide. This inflammatory disease continues to cause significant premature mortality and morbidity (Doyle et al., 2014 ▶; Zyrianova et al., 2006 ▶).

Rheumatoid arthritis has unknown etiology but scientists believe that immunological and nutritional factors, oxidative stress, hormonal agents, genetics, and infection disease are contributed in occurrence of the disease. Monocytes, macrophages, and T and B cells lymphocytes are involved in pathogenesis of RA with increasing production of inflammatory cytokines such as tumor necrosis factor (TNF-α), interleukin (IL)-1β, and IL-6 which some of these cells lead to destructive progress of synovial cartilage and bones (Goronzy et al., 2006 ▶; Weyand et al., 2000 ▶). TNF-α stimulates nuclear factor kappa B (NF-κB) signaling pathway that leads to production of inflammatory cytokines which play an important role in RA (El Gazzar et al., 2000 ▶). Three main immunoregulatory strategies in RA include inhibition of TNF-α, shifting immune response from Th1 that produces pro-inflammatory cytokines to Th2 that produces anti-inflammatory cytokines such as IL-10, and alteration immune responses toward IL-10 which is able to induce a skew from inflammatory to anti-inflammatory reaction (Yudoh et al., 2000 ▶).

Oxidative stress can cause cell death by damaging cellular lipids, proteins, and DNA and results in many diseases in particular autoimmune diseases. Imbalance between increased exposure to free radicals and antioxidant defenses is also a highlighted feature of many acute and chronic diseases. Under the oxidative stress conditions, an endogenous antioxidant, such as superoxide dismutase (SOD), catalase (CAT), and glutathione may fail to counter reactive oxygen species (ROS) (Ebru et al., 2008 ▶).Biomarkers of oxidative stress have been widely used to evaluate the relation between oxidative damage to macromolecules (lipids, DNA, and proteins) and disease progression (Wadley et al., 2014 ▶). Elevated serum malondialdehyde (MDA), a biomarker of lipid peroxidation, together with decreased activities of antioxidants including CAT, SOD, and glutathione peroxidase have been reported in RA patients as compared to healthy individuals (Kurien et al., 2003 ▶).* Nigella sativa*, commonly known as black cumin or black seed belongs to the plant family of Ranunculaceae and has long been used in Iranian traditional medicine for treating various disorders and conditions pertaining to respiratory system, digestive tract, kidney and liver function, cardio vascular system, and immune system support, as well as for general well-being (Amin et al., 1991 ▶). Most of the therapeutic effects of this plant are due to thymoquinone (TQ) which is the main active ingredient in the oil of this plant. It is known that plant possess broad anti-inflammatory activities on several inflammation-based models such as RA, colitis, and peritonitis (Ahmad et al., 2013 ▶). In animal models of inflammation, this compound suppresses the elevated levels of pro-inflammatory cytokines and pro-oxidants (El-Mahmoudy et al., 2005 ▶). Thus, the current randomized clinical trial was conducted to investigate the effects of *Nigella sativa* oil on selected inflammatory cytokines and oxidative stress status in women patients with RA.

## Materials and Methods

Study design and Subjects

The study was a randomized, double-blinded, placebo-controlled clinical trial. The ethics committee of Tabriz University of Medical Sciences (TBZMED), Tabriz, Iran provided ethics approval, and the trial was registered on the Iranian Registry of Clinical Trials website, which is available at(http://www.irct.ir/, IRCT: IRCT2012120811689N1).

A sample size of 18 patients per group was calculated based on previously published papers. Collectively, total of 25 patients were included in the study for each group to cover 30% dropout (Toubiet al., 2005 ▶).Patients were recruited from the Sheykholrayis outpatient Clinic affiliated to TBZMED from May 2012 to November 2013.

The inclusion criteria consisted of (1) volunteer women patients aging between 20 to 50 with mild to moderate RA, according to 2010 ACR-EULAR criteria, (2) being under treatment with methotrexate, hydroxychloroquine, and prednisolone less than 10 milligrams per day (DMARDs), (3) not receiving any non-steroidal anti-inflammatory drugs or cytokine inhibitors with stable medication for at least 2 months prior to the intervention, and (4) having body mass index (BMI) less than 40. The exclusion criteria of the study included those with (1) pregnancy and lactating, (2) hormone therapy or receiving oral contraceptives, (3) having any metabolic disorders such as diabetes mellitus, lactose intolerance, Cushing's syndrome, or thyroid dysfunctions, (4) kidney or liver diseases, (5) chronic inflammatory diseases including inflammatory bowel diseases, and (6) any history of taking antioxidant or anti- inflammatory supplements 4 weeks prior to the interventions or being on weight reduction diets and smoking.

Intervention group received two 500 milligram capsules containing* Nigella sativa*oil each day (Ghetaet al., 2011 ▶) for 8 weeks. The other group consumed two placebo capsules (paraffin) per day for the same period of time (120 capsules per bottle, produced by Barij Asans of Kashan, Kashan, Iran). *Nigella sativa *oil and placebo were dispensed in capsules with identical appearance to ensure double blindness of the study. Besides, *Nigella sativa* oil and placebo capsules were encoded by another person blinded to the study.

At baseline and at the endpoint of the study, weight and height were measured and BMI was calculated. Moreover, demographic information (such as age, job, disease history, medication, and marital status), physical activity level, and psychological stress of the patients were assessed using IPAQ and STAI-Y questionnaires, respectively. Moreover, dietary intake of the participants was evaluated using a three-day dietary record before and after the intervention. Dietary data were analyzed using Nutritionist IV software (First Databank, San Bruno, CA, USA).

In order to establish compliance with treatment, all of the participants were asked to keep unused capsules in drug containers. All of the patients were monitored by phone call every 15 days for any probable adverse events.

**Blood sampling**

At the baseline and endpoint of the study, after 10-12 hours fasting, venous blood was drawn and centrifuged for 15 minutes at 1500 g to obtain serum. Serum samples were stored at -20 ºC until biochemical analysis.

**Measurement of cytokines levels**

Serum levels of TNF-α and IL-10 were determined using commercially available cytokine ELISA kits (DIASource, Belgium and eBioscience, USA, respectively) following instructions of the manufacturers at 450 nM wavelength in an ELISA plate reader apparatus (Awareness, Statfax-2100 model, USA). 

**Measurement Antioxidant defense system (TAS, SOD, and catalase)**

Serum total antioxidant capacity (TAC) was determined using a Randox TAS kit (Randox Laboratory, UK) (Miller et al., 1993 ▶). Superoxide dismutase (SOD) activity was measured spectrophotometrically using a Ra nsod kit (Randox Laboratory, UK). Catalase activity (CAT) was measured using the method proposed by Aebi (Aebiet al., 1984 ▶). All tests were performed with an automatic analyzer (Abbott model Alcyon 300, USA). 

**Measurement of oxidative stress**

Nitric oxide (NO) was measured using the method proposed by Griess (Moshageet al., 1995 ▶). Serum MDA concentration was determined using the thiobarbituric acid method described by Bilici (Bilici et al., 2001 ▶).

**Statistical analysis**

The data were analyzed using SPSS version 16 (SPSS Inc., Chicago, IL, USA). Value of pless than 0.05 was considered significant. The quantitative and qualitative data were presented as mean ± SD and median (25^th^ and 75^th^ percentiles), respectively. The normality of variables and homogeneity of variances were tested using the Kolmogorov–Smirnov and Leven tests, respectively. Chi-squared test was used to compare the two groups for background characteristics. Independent t-test or Mann–Whitney U tests were used to compare mean values between groups at baseline. Mean values before and after the study period were compared within the groups using the paired t-test or Wilcoxon test. Analysis of covariance (ANCOVA) was used to compare the two groups for the measures at the end of the study after adjusting for the baseline measures and covariates (i.e., changes in BMI and state and trait anxiety scores throughout the study as well as menopausal status). 

## Results

**Subjects**

**Figure 1:**
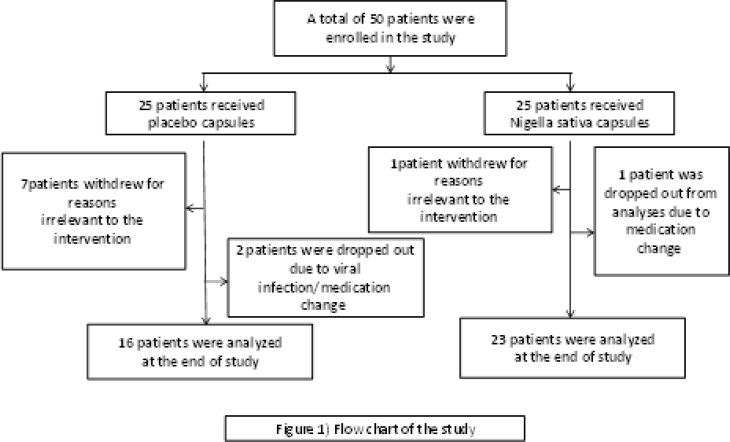
Flow diagram of the progress through the phases of randomized trial of two groups

Fifty female patients with RA were recruited in the present clinical trial and 42 women completed the study. Eight percent (8%) of the *Nigella sativa* group and 20% of placebo group did not complete the 8-week treatment course (Figure 1). No severe adverse effects of the treatment were reported.

**Patients' characteristics**

Baseline characteristics of the patients are presented in Table 1. Baseline characteristics of the patients did not differ between the two group

**Table 1:** Baseline characteristics of the studied subjects

	**Placebo group (n = 16) **	**Nigella sativa group (n = 23)**	**P**
**Age (y) **	40.58 ± 8.6*	43.90 ± 5.23*	0.27†
**BMI (kg/m** ^2^ **) ** **Menopausal status** ‡	24.35 ± 3.38*	24.93 ± 6.22*	0.70†
**Premenopausal**	13 (68.40)	16 (69.60)	0.75†
**Postmenopausal** **Physical activity**	6 (31.60)	7 (30.40(	
**Low **	11 (68.8)	18 (78.3)	
**Moderate**	5 (31.2)	5 (21.7)	0.40††
**High**	0 (0.0)	0 (0.0)	
**State anxiety** ‡			
**No or minimum**	2 (12.5)	6 (26.2)	
**Mild**	10 (62.5)	12 (53.2)	0.11††
**Moderate **	3 (18.8)	4 (17.3)	
**Severe**	1 (6.2)	1 (4.3)	

† Independent Student t-test for age and BMI; Chi-squared test for menopausal status.

†† Mann-Whitney U test.

* mean ± SD.

‡ Frequency (percent)

**Table 2:** Energy and macronutrient intake in the two experimental groups at baseline and throughout the study.

		**Placebo group (n = 16) **	**Nigella sativa group (n = 23)**	**p-value**
**Energy (cal)**			
Baseline		1742.68 ± 471.49	1889.82 ± 564.32	0.21*
End of study		1692.41 ± 523.30	1894.82 ± 548.17	0.90**
p-value***		0.89	0.76	
**Protein (g**)				
Baseline		56.35 ± 14.73	58.86 ± 15.80	0.43*
End of study		59.00 ± 14.23	60.21 ± 14.17	0.38**
p-value***		0.61	0.81	
**Fat (g)**				
Baseline		55.06 ± 17.25	62.60 ± 11.12	0.27*
End of study		56.85 ± 18.13	60.80 ± 14.02	0.69**
p-value***		0.17	0.18	
**PUFAs (g)**				
Baseline		14.37 ± 7.07	16.36 ± 4.15	0.39*
End of study		15.37 ± 5.59	15.86 ± 7.61	0.91**
p-value***		0.12	0.63	
**MUFAs (g)**				
Baseline		19.02 ± 7.05	20.58 ± 5.07	0.70*
End of study		19.34 ± 7.81	19.83 ± 5.67	0.91**
p-value***		0.19	0.55	
**SFAs (g)**				
Baseline		14.43 ± 6.14	17.15 ± 7.10	0.14*
End of study		14.52 ± 6.51	17.98 ± 5.46	0.53**
p-value***				
**Fiber (g)**				
Baseline		12.12 ± 3.82	12.18 ± 3.90	0.95*
End of study		12.52 ± 2.78	13.26 ± 5.56	0.40**
p-value***		0.95	0.58	

* mean ± SD are presented for the measures**.**

* Independent Student t-test.

** Based on analysis of covariance adjusted for baseline measures.

*** Paired Student t-test**.**

At the end of the study, there was no statistically significant difference in BMI value between or within the groups. There were no differences between the two groups at the beginning of study regarding to doses and types of drugs. STAI-Y scores calculated at the beginning of the intervention and physical activity level showed no significant difference between the two groups in baseline of the study.

The patients’ micronutrient intakes in the two groups remained stable during the study period. Analysis of dietary questionnaires, summarized in Table 2, indicated that there were no significant differences in energy and macronutrient intakes at baseline and end of study course in both of the groups.

Disease activity (DAS28) score was not significantly different between the two groups at baseline. At the end of the study, DAS28 score significantly decreased in the *Nigella sativa* group (p<0.05), but it did not change significantly in the placebo group. The DAS28 score was significantly lower in the *Nigella sativa* group as compared with the placebo group at the end of the study.

**Biochemical data**

As shown in Table 3, in the *Nigella sativa* group, serum IL-10 was significantly increased at the end of the study in comparison with the baseline (p<0.01).

**Table 3:** Effect of *Nigella sativa* as compared with placebo on inflammatory, antioxidant, and stress oxidative biomarkers in female patients with rheumatoid arthritis

	**Placebo group (n=16) **	**Nigella sativa group (n =23)**	**p-value**
**TNF-α (mg/L) **			
Baseline	12.20 (7.82,15.06)	13.29 (8.19, 17.30)	0.65**†**
End of study	12.20 (9.04,18.69)	9.42 (5.72, 14.83)	0.27**
Mean difference (95% CI)	0 .00 (-3.47, 6.79)	0.15 (-7.87, 4.48)	
p-value***	0.72	0.74	
**IL-10**			
Baseline	5.55 (1.90, 20.55)	7.80 (2.40, 12.20)	0.84†
End of study	7.8 (0.60, 43.50)	11.30 (3.50, 53.70)	0.34**
Mean difference (95% CI)	0.40 (-1.62, 22.95)	8.90 (1.50, 45.90)	
p-value***	0.21	0.009	
**SOD**			
Baseline	1221.80 ± 451.44	1353.6 ± 449.99	0.37*
End of study	1362.50 ± 444.64	1577.6 ± 579.31	0.20**
Mean difference (95% CI)	89.08 (-121.10, 476,48)	214.59(-400.40, 769.73)	
p-value***	0.21	0.13	
**Catalase**			
Baseline	122.28 ± 46.36)	124.72 ± 35.67	0.86*
End of study	116.03 ± 26.40)	118.13 ± 26.67	0.81**
Mean difference (95% CI)	-0.84 (-33.25, 10.57)	-5.15 (-25.09, 3.23)	
p-value***	0.64	0.12	
**TAC**			
Baseline	1.37 ± 0.26	1.34 ± 0.27	0.76*
End of study	1.33 ± 0.19	1.47 ± 0.29	0.09**
Mean difference (95% CI)	-0.06(-0.15, 0.04)	0.09(0.05, 0.38)	
p-value***	0.51	0.06	
**MDA**			
Baseline	3.06 ± 1.20	3.07 ± 0.88	0.97*
End of study	3.01 ± 0.73	2.66 ± 0.83	0.18**
Mean difference (95% CI)	0.10(-0.80, 1,10)	-0.60(-1.10, 0,20)	
p-value***	0.94	0.03	
**No**			
Baseline	71.75 ± 53.26	104.70 ± 62.16	0.08*
End of study	90.20 ± 58.94	61.83 ± 43.85	0.12**
Mean difference (95% CI)	13.00(-37.00, 103.00)	-47.00 (-79.00, -4.00)	
p-value***	0.40	0.013	

TNF-α: Tumor necrosis factor-alpha; IL-10: Interlukin-10; SOD: Superoxide dismutase; TAC: Total antioxidant capacity; MDA: *Malondialdehyde*; NO: Nitric oxaid. mean ± SD and median (percentiles 25 and 75) are presented for normally and not normally distributed measures, respectively. Median of differences (percentiles 25 and 75) are presented instead of mean difference (95% CI). †Mann-Whitney U test

** Based on ANCOVA adjusted for baseline measures and confounding factors (duration)

††† Wilcoxon test

* Independent t-test

*** Paired t-test

At the end of the study, analysis of covariance did not show any significant differences in serum level of IL-10 in the two groups. Malondialdehyde and NO were significantly decreased compared to the baseline values in the intervention group (p=0.04 and p=0.01, respectively). However, there was no statistically significant difference between the two groups at the baseline or end of the study. Moreover, our study showed no significant within- and between-group changes in levels of TNF-α, SOD, catalase, and TAC after the intervention (p>0.05).

## Discussion

Existing evidence indicates that chronic oxidative and/or inflammatory state plays a substantial role in the pathogenesis of RA (Katsoulis et al., 2003 ▶). Therefore, anti-inflammatory and/or anti-oxidant interventions may provide a useful approach to attenuate disease progression.

The results of the present study showed that eight weeks of *Nigella sativa* supplementation significantly increased anti-inflammatory cytokine (IL-10) and reduced non-significantly pro-inflammatory cytokine, TNF-α. The findings are in consistence to earlier studies (Majdalawieh et al., 2010 ▶; Umar et al., 2012 ▶). Previous studies on the pharmacological effects of *Nigella sativa* seed and TQ confirmed multiple benefits including suppression of pro-inflammatory cytokines, pro-oxidants and elevation of some anti-inflammatory cytokines such as IL-10 in animal models with inflammatory (Umar et al., 2012 ▶). 

Findings of a study on animal model of RA indicated that oral administration of TQ resulted in significantly reduced levels of TNF-α and increased level of IL-10 (Umar et al., 2012 ▶). In another study, it has also been shown that treatment with TQ led to a reduction of TNF-α in experimentally-induced arthritis in rats (Tekeoglu et al., 2006 ▶). The results of an in-vitro study showed that the secretion of TNF-α and NO was significantly suppressed by aqueous extract of *Nigella sativa* indicating that anti-inflammatory effects of the extract of this plant (Majdalawieh et al., 2010 ▶).A link between inflammation and bone homeostasis has been attributed to the effects of cytokines such as IL-1β, TNF- α, expressed in patients with RA and in the arthritic joints of rat with collagen-induced arthritis (Umar et al., 2012 ▶; Schett et al., 2008 ▶).

It is suggested that blockade of these molecules results in reduction of disease severity and bone resorption (Schett et al., 2008 ▶; Williams et al., 2004 ▶). In contrast, IL-4 and IL-10 have potent anti-inflammatory effects and suppress cartilage and bone pathology in RA (Juarranz et al., 2005 ▶). Treatment with TQ shifts the balance of cytokines toward a bone protecting pattern and reduces production of free radicals (Majdalawieh et al., 2010 ▶; Wu et al., 2011 ▶).

Pro-oxidants (free radicals) and defects of anti-oxidant (scavenging) mediators have important roles in pathogenesis of RA and are key initiators in tissue injury observed in the patients (Schulz et al., 2000 ▶). Nitric oxide (NO) is an important signaling molecule, produced as part of the inflammatory response from activated cells and macrophages (Seo et al., 2001 ▶). Inflammatory cytokines in chondrocytes are known to facilitate high amounts of NO implicated in apoptosis of chondrocytes (Blanco et al., 1995 ▶). 

In the present study, treatment with *Nigella sativa* produced a significant reduction of NO and MDA levels.The results were consistent with outcomes provided by previous studies indicating TQ may reduce nitrite production, a parameter for NO synthesis, and decrease both gene expression and protein synthesis levels of iNOS (Umar et al., 2012 ▶; El-Mahmoudy et al., 2002 ▶). It has been reported that TQ supplementation prevents the development of diethylnitrosamine-induced initiation of liver cancer in rats by decreasing oxidative stress biomarkers such as NO (Sayed-Ahmed et al., 2010 ▶). In another study on rabbits, it has been shown that *Nigella sativa *has hepatoprotective effects against Isoniazid-induced hepatotoxicity, partly by reduction of MDA (Hassan AS et al., 2012 ▶). *Nigella sativa* supplementation reverses osteoporosis in ovariectomized rats in part by reduction of TNF-alpha and MDA (Seif AA, 2014 ▶). 

Unexpectedly, in the present study *Nigella sativa* did not change serum levels of SOD, CAT, and TAC. It is highly possible that this contradiction between animal model studies and our study rose from lack of administration of anti-inflammatory drugs in animals during those interventions, dose differences, or different experimental conditions. 

The suggested mechanism that *Nigella sativa* may affect both oxidative stress and inflammatory process simultaneously is through inhibition of NF-κB (Wilkins et al., 2011 ▶). Thymoquinone inhibits nuclear expression of NF-κB p65 subunit and inhibits in-vivo binding of p50 subunit to TNF-α promoter (El Gazzar et al., 2007 ▶). TNF-α, IL-6, and a variety of other cytokines are not only up-regulated by NF-κB, but also act as activators of NF-κB leading to perpetuation of pro-inflammatory condition (Ahn et al., 2005 ▶). On the other hand, ROS is known as a considerable cause for tremendous oxidative stress in RA and plays an essential role for both upstream and downstream pathways of NF-κB (Ishibashi et al., 2013 ▶). Hence, *Nigella sativa* may likely interrupt these interactions via suppression of NF-κB and plays an important part in its anti-oxidant/anti-inflammatory activity (Woo et al., 2012 ▶).

This study demonstrated that *Nigella sativa* could improve inflammation and reduce oxidative stress in patients with RA and suggested that supplementation with an extract of the seed may be a beneficial adjunctive therapy in this population of patients. We believe that our results will contribute to the clinical application of *Nigella sativa* in management of patients with RA. 

## Conflict of interest

The authors declare that they have no conflict of interests.
